# Focal IgG4-related autoimmune pancreatitis with distal choledochal adenocarcinoma: a rare case report

**DOI:** 10.1186/s12876-021-01996-y

**Published:** 2021-11-10

**Authors:** Han Wang, Lan Yao, Ligang Wang, Xixi Sun, Bin Huang

**Affiliations:** 1 The Fourth Clinical Medical College, Zhejiang Chinese Medical University, Hangzhou, 311402 Zhejiang Province China; 2 The Second Clinical Medical College, Zhejiang Chinese Medical University, Hangzhou, 311402 Zhejiang Province China; 3 Department of Ultrasonography, Zhejiang Provincial People’s Hospital, Hangzhou, 310014 Zhejiang Province China; 4 Ultrasound Department, Zhejiang Hospital affiliated to Zhejiang University School of Medicine, No. 1229 Gudun Road, Xihu District, Hangzhou, 310013 Zhejiang Province China

**Keywords:** IgG4, Autoimmune pancreatitis, Carcinoma of bile duct, IgG4-related disease, Case report

## Abstract

**Background:**

Autoimmune pancreatitis (AIP) is a rare disease that manifests as pancreatic involvement in systemic IgG4-related disease (IgG4-RD), a special type of chronic pancreatitis caused by autoimmune abnormalities. The main imaging manifestations of IgG4-related AIP consist of diffuse or localized pancreatic enlargement and irregular pancreatic duct narrowing. The diagnosis of AIP is challenging because it can present with focal lesions, similar to radiologically bile duct cancer or pancreatic cancer.

**Case presentation:**

A 55-year-old male patient was admitted with painless jaundice and multiple radiographic findings of pancreatic head mass, as well as intrahepatic and extrahepatic bile duct dilatation. Various imaging methods indicated pancreatic cancer. However, the endoscopic ultrasonography guided fine needle aspiration (EUS-FNA) and a laparoscopic pancreatic biopsy suggested an IgG4-related AIP. After one month, magnetic resonance imaging showed that the lesion had slightly grown. Combined with CA19-9 and other indexes, the possibility of malignancy was high and there were still surgical indications. The pathological analysis following a pancreaticoduodenectomy revealed poorly differentiated adenocarcinoma in the distal common bile duct.

**Conclusion:**

To date, few reports have described pancreatic or extrapancreatic malignancies in AIP patients, and no association between AIP and bile duct adenocarcinoma has been previously confirmed. This case discuss the differentiation between AIP and malignancy, recent research progress, and the correlation between the two diseases, highlights the importance of carefully evaluating patients with AIP to rule out potential tumors, as well as the critical need for follow up treatment.

## Background

Autoimmune pancreatitis (AIP) was originally described as primary inflammatory sclerosis of the pancreas by Sarles et al. [1] in 1961. Until 1995, Yoshida and others considered AIP as a diagnostic entity. AIP is a rare type of chronic pancreatitis that can be divided into two clinical types: type 1 and type 2 AIP [2]. Type 1 AIP, also known as lymphoplasmacytic sclerosing pancreatitis, is considered to represent a pancreatic manifestation of systemic IgG4-RD. The clinical features include elevated serum IgG4 levels and a favorable response to steroids. In type 2 AIP, IgG4 levels are not elevated, indicating the presence of neutrophil infiltration.

The imaging features of AIP consist of irregular stenosis of the pancreatic duct, the presence of autoantibodies, and diffuse pancreatic swelling [3, 4]. In rare cases, AIP can appear as a focal mass, similar to pancreatic cancer [5, 6]. Since the two treatments are essentially different, it is important to make a correct differential diagnosis between the two diseases. Since the relationship between AIP and cancer remains unclear, once autoimmune pancreatitis is diagnosed, it should not be slacking off, since malignant lesions may also be found.

Here, we report a diagnostically challenging case, in which a patient was found to have an occupying pancreatic head. The patient appeared to have both focal mass type IgG4-associated pancreatitis and adenocarcinoma of the lower common bile duct. Since cancer in AIP patients is considered to be rare, no association between AIP and bile duct adenocarcinoma has been previously confirmed. To date, few reports have described pancreatic or extrapancreatic malignancies in AIP patients. It is important to discuss the association of malignant tumors in AIP patients to improve the clinical understanding of this disease and discuss the differentiation between AIP and malignancy, recent research progress, and the correlation between the two diseases.

## Case presentation

**Fig. 1:**
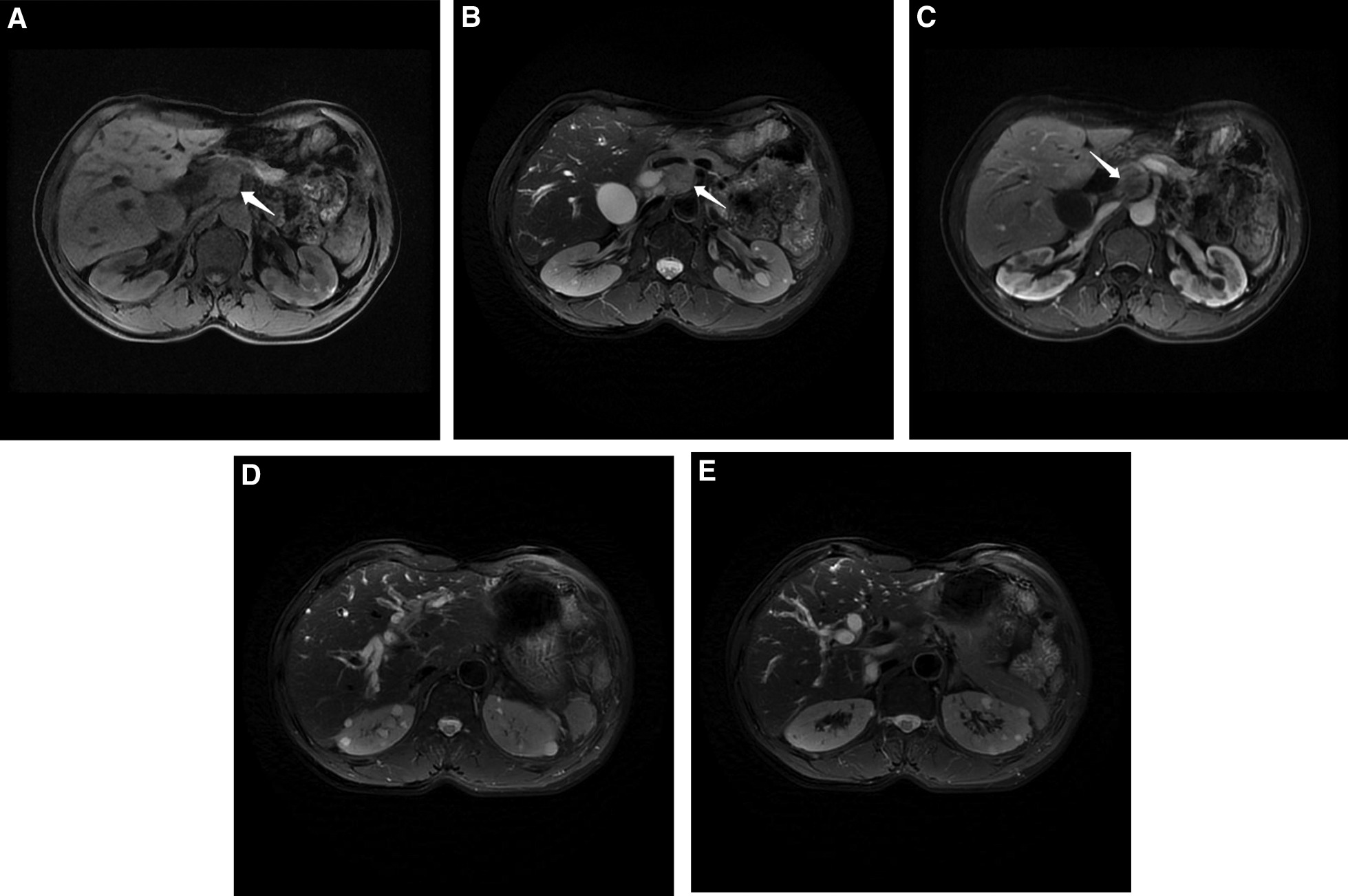
Enhanced magnetic resonance imaging. **a** T1WI showed low signal intensity of a pancreatic head mass. **b** On T2WI, the mass of the head of the pancreas shows iso-signal intensity, and the common bile duct, abdominal trunk, and other branches were surrounded. **c** MR-enhanced pancreatic head mass showed uneven enhancement. **d** Intrahepatic bile duct dilatation. **e** No significant swelling or distal pancreatic duct widening was observed in the body and tail of the pancreas

A 55-year-old male patient with yellow staining of the skin and sclera for 2 weeks. No obvious abnormalities were observed upon admission. The patient underwent a prior splenectomy. There was no other notable history of past illness. The initial laboratory tests showed 340 U/L alanine aminotransferase, 73.5 μmol/L total bilirubin, 96.9 U/mL carbohydrate antigen 19–9(CA19-9), 3.74 g/L IgG4, and 327 U/L plasma total amylase. The abdominal computed tomography (CT) showed occupancy of the pancreatic head with dilatation of the intrahepatic and extrahepatic biliary ducts and enlargement of the gallbladder. Enhanced magnetic resonance (MR) examination revealed a mass with an abnormal signal shadow, low signal on T1WI, an equal signal on T2WI, a slightly high signal on diffusion weighted imaging, and uneven enhancement, which was lower than the normal pancreatic tissue. The celiac trunk and its branches were surrounded, and both the intrahepatic and extrahepatic bile ducts had expanded like soft vines (Fig. 1).

**Fig. 2:**
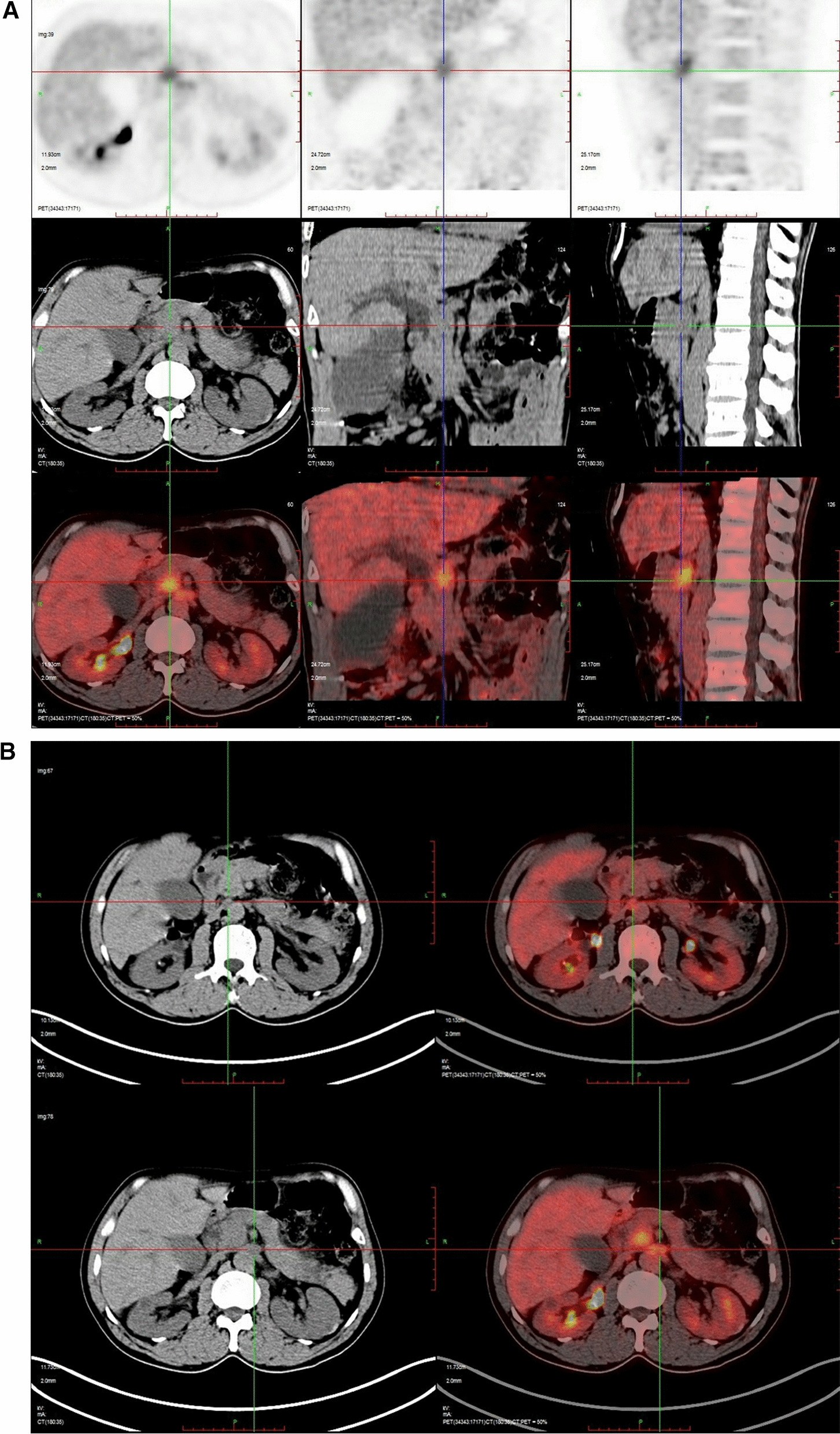
Positron emission computed tomography findings. **a** The round mass of the head of the pancreas exhibited posterior protrusion, with increased tracer distribution (SUVmax 6.91). The lesion involved the adjacent common bile duct and no abnormality was observed in the remaining pancreas. **b** Two abnormal perfusion foci in the retroperitoneum (behind the head of the pancreas) were closely attached to the abdominal aorta and left renal vein (SUVmax 4.45)

**Fig. 3:**
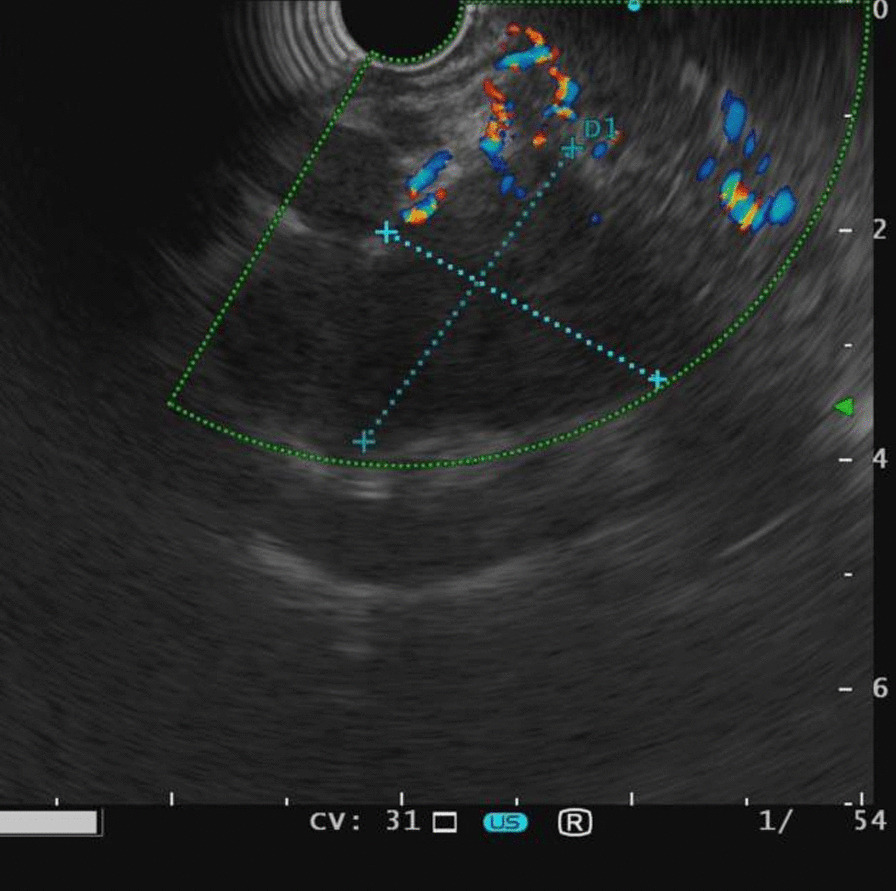
Endoscopic ultrasonography demonstrated an irregular hypoechoic area within the head of the pancreas above, involving the lower common bile duct

**Fig. 4:**
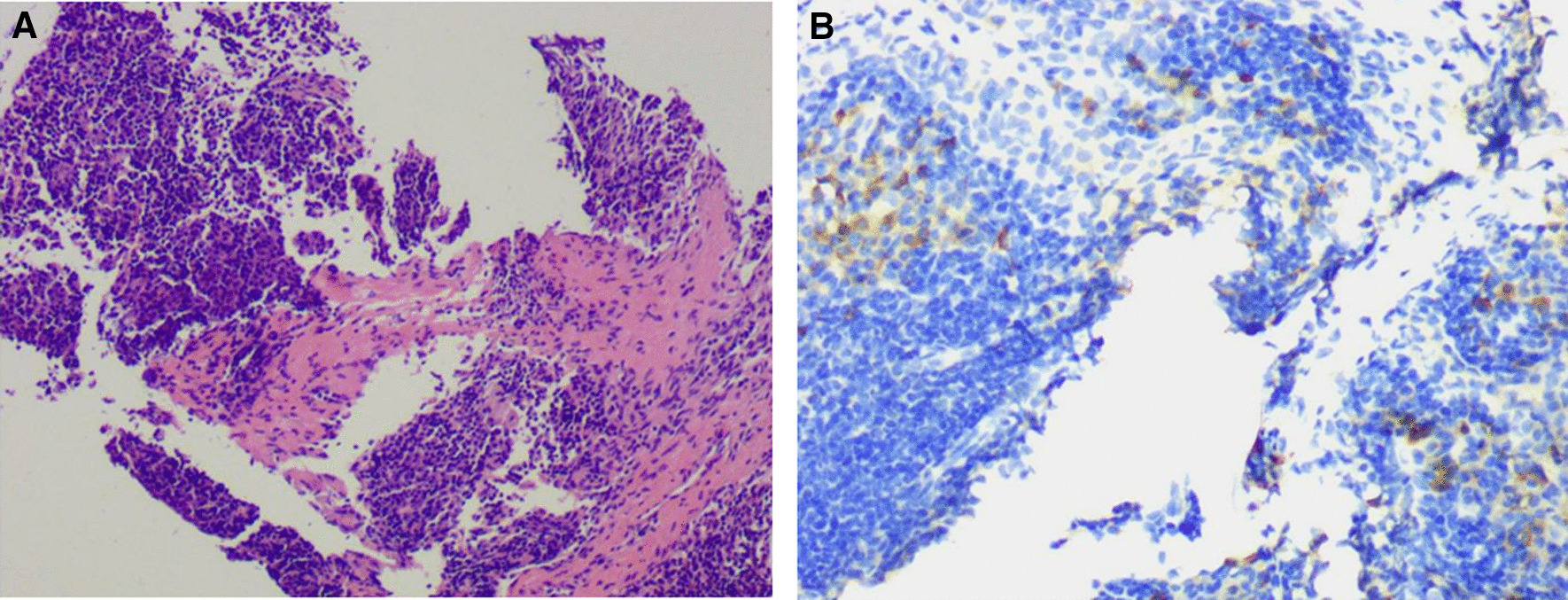
Histological findings of tumor-forming pancreatitis without a background of typical chronic pancreatitis. **a** Tumor lesions manifested as lymphoid hyperplasia, germinal centers, lymphoplasmacytic infiltration, and interlobular and intralobular fibrosis. Hematoxylin and Eosin (HE), original magnification: 100 ×. **b** Immunohistochemical study showing positive immunoreactivity for IgG4 in the plasma cells. More than 90 IgG4-positive plasma cells/high power field were detected. Original magnification: 200 ×

The positron emission tomography-CT (PET-CT) examination revealed a low-density lesion in the head of the pancreas, approximately 2.3 cm 2.1 cm in size, locally protruding behind the head of the pancreas, and increased FDG metabolism (SUVMAX 6.91). Pancreatic cancer was considered and the lesion involved the adjacent common bile duct. There were two retroperitoneal metastatic foci located posterior to the head of the pancreas (Fig. 2). EUS showed an irregular hypoechoic mass in the uncinate process of the pancreas with irregular borders, invading the lower common bile duct with an inner diameter of 9 mm. The superior mesenteric artery was immediately adjacent to the mass (Fig. 3). Then laparoscopic puncture histopathology showed fibrous tissue proliferation with a large number of plasma cells and lymphocyte infiltration in pancreatic tissue. This finding when combined with the immunohistochemistry results was consistent with IgG4-related autoimmune pancreatitis (IgG4 about 90 cells/high power field (HPF)); no cancer cells or pancreatic intraepithelial neoplasia were observed (Fig. 4). The patient was given prednisone 5 mg qd as long-term steroid treatment in the following month, then discharged from the hospital.

**Fig. 5:**
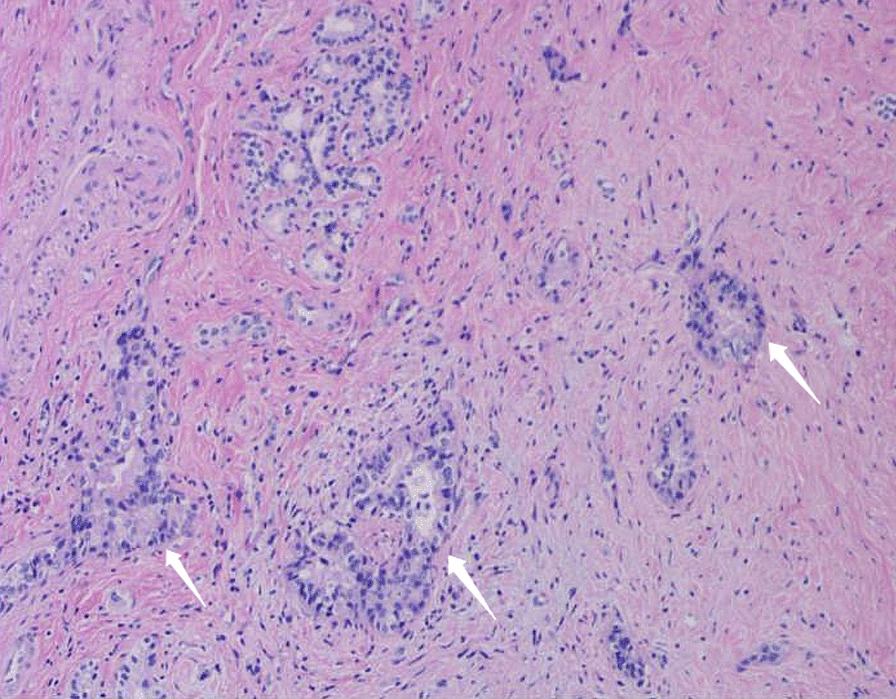
Histological findings of the pancreaticoduodenectomy specimen. Poorly differentiated common bile duct adenocarcinoma infiltrating nerve. HE, original magnification: 100 ×. Immunohistochemical results: CEA +, CK7 +, Ki67(40% +), CDX2(-), Muc-1(+)

After one month, the patient was admitted to the hospital again because the enhanced MR examination revealed a mass in the head of the pancreas, which appeared larger. The patient was deemed to still have indications for surgery and a laparoscopic pancreaticoduodenectomy + laparoscopic retroperitoneal lymph node dissection + intestinal adhesion release was performed. During the operation, a hard mass was observed in the upper part of uncinate process of pancreas near the root of abdominal trunk, which invaded the pancreatic segment of bile duct and the bile duct was dilated with a diameter of 1.2 cm. The lesion did not involve the main pancreatic duct, which was about 0.2 cm wide. The head of the pancreas was seen to be edematous and lobulated. After resection of the lesion, anastomoses pancreaticum using Blumgart anastomosis. The operation lasted 10 h and 400 ml of bleeding. Routine pathology after surgery revealed a poorly differentiated adenocarcinoma in the lower segment of the common bile duct wall, approximately 1.7 × 1.5 × 1.0 cm in size, with positive nerve invasion and indicated metastatic adenocarcinoma of the abdominal trunk root mass (Fig. 5). Four of the 16 regional lymph nodes showed metastasis. Vascular involvement was negative, major duodenal papilla and pancreatic duct were not involved, and all resection margins were negative for tumor. A large amount of lymphocyte infiltration was observed in pancreatic tissue, and no obvious intraepithelial neoplasia was observed. The patient had diarrhea after operation, the rest of the general situation was good, and was discharged 10 days after operation. He was treated with the mFOLFRINOX regimen postoperative chemotherapy. A follow-up CT scan at 4 months post-surgery confirmed that the patient was in remission. During the postoperative follow-up, the total amylase dropped to the normal range at about 10d after operation, the C-reactive protein dropped to the normal range at about 3 months after operation, and the plasma IgG4 levels increased at about 2 months after operation ( 3.41 g / L). CEA and CA19—9 have gradually increased since the follow-up.

## Discussion and conclusions

IgG4-related disease (IgG4-RD) has only recently been discovered and was not internationally recognized until 2011 [7]. Ig4-RD is characterized by dense lymphocyte plasma cell infiltration, storiform fibrosis, and occlusive phlebitis involving multiple organs. The main clinical manifestation of this disease is observed in the pancreas, and approximately 45% occur in extra-pancreatic tissues. AIP was first reported by Yoshida et al. [8], which conformed to the definition of autoimmune disease to involve hyperimmunoglobulinemia, positive serum autoantibodies, and a response to steroid treatment. In 2011, the International Association of Pancreatology developed an international consensus diagnostic standard for AIP [9], which is considered to be the most authoritative standard for the diagnosis of AIP and has been widely recognized. AIP is a specific type of pancreatitis for which the clinical symptoms, laboratory tests, and imaging findings are similar to those of pancreatic cancer, malignant lymphoma, and other types of pancreatitis due to a lack of specific clinical manifestations. Therefore, the differential diagnosis must be carefully carried out.

AIP is associated with specific imaging findings, including pancreatic enlargement and irregular narrowing of the main pancreatic duct; serological abnormalities (e.g., elevated immunoglobulin levels or the presence of autoantibodies); and histological features, including lymphocytic infiltration and pancreatic interstitial fibrosis. AIP often presents as obstructive jaundice, which is a partial narrowing of the bile duct pancreas due to exogenous compression of the inflamed pancreatic head and inflammatory changes in the common bile duct itself. There can also be other complex extrapancreatic manifestations, such as sclerosing cholangitis, sialadenitis, and retroperitoneal fibrosis. In addition, oral prednisolone (PSL) therapy has been shown to have a significant beneficial effect on acute pancreatitis [10].

A study by Hamano et al. [11] reported that the serum concentration of IgG4 was significantly and specifically increased in patients with AIP; however, not all pancreatic masses with elevated IgG4 expression were AIP. Studies have shown that serum IgG4 elevation and IgG4-positive plasma cell infiltration can also possibly occur in pancreatic cancer and some extrapancreatic cancers. Moreover, increased levels of IgG4 expression alone cannot rule out the existence of cancer; however, the level of IgG4 expression in the pancreas is generally low (serum IgG4 levels do not typically exceed 2 the normal upper limit, and tissue IgG4-positive cells are often < 50/HPF [12–14]. Therefore, it is helpful to distinguish AIP from cancer and avoid unnecessary surgery by measuring the IgG4 titer.

Some scholars have reported AIP in association with a focal mass [5, 6], which makes it more difficult to distinguish AIP from a pancreatic malignant tumor. The study by Chari et al. [15] retrospectively compared 48 patients with autoimmune pancreatitis with obstructive jaundice and 100 patients with pancreatic cancer, and suggested the following American strategy: any patients with pancreatic cancer with high imaging characteristics should be treated as cancer. When the imaging features highly suggest or point to cancer elimination, the above strategies do not depend on a tissue diagnosis or needle aspiration biopsy. In our patient, the CT scan revealed a mass in the focal low density center, and EUS also showed a measurable mass in the pancreatic head. Although the evidence of a histological biopsy was insufficient, the influence of a tissue sampling error could not be ruled out. High levels of CA 19–9 and the infiltration and metastasis of peripheral tissues revealed by imaging suggest a malignant tumor, and aids in the decision of whether to operate.

When IgG4-RD is accompanied by a malignancy, the malignancy may occur in multiple organs [16], which can occur before, during, or after the diagnosis of IgG4-RD. The sequence of the two diseases remains uncertain. Both the synchronous and atypical occurrence of AIP and pancreatic tumors have been reported. In AIP patients, the incidence of pancreatic tumors is approximately 0.1–4.8% [17, 18]. This co-occurrence suggests that a definite diagnosis of AIP does not rule out the co-presence of pancreatic tumors.

The perspective that IgG4-RD is easily associated with malignant tumors remains controversial [19]. The study by Shiokawa et al. [20] performed a multicenter retrospective cohort study which showed that the cancer incidence in AIP patients was significantly higher than that of the standard population matched by gender, age, and observation period. This supports the view that AIP is a strong risk factor for cancer, and suggests an attractive concept that AIP type 1 and IgG4-RD can occur as a paraneoplastic syndrome. Wallance et al. [21] conducted a case–control study in 2016 that suggested that both malignancies with IgG4-RD and/or AIP may increase the risk of cancer, as well as preexisting malignancies associated with subsequent development of IgG4-RD and/or AIP. Moreover, Kamisawa et al.[22] reported an association between a mutation called KRAS in the epithelial cells of the pancreas, common bile duct, and gallbladder in patients with AIP. Therefore, the authors suggest that AIP may represent a risk factor for pancreatic cancer. The presence of both IgG4-AIP and cholangiocarcinoma in our patient's pancreatic lesions combined with previous reports of IgG4-associated disease associated with malignancy, suggests a significant pathophysiological association between at least these two entities.

Some studies have failed to establish a link between AIP and cancer. Hirano et al. [23] analyzed 113 patients with IgG4-RD, including 95 patients with AIP, and determined the incidence of cancer during patient follow-up. Although 14 patients (12.4%) were diagnosed with cancer during the follow-up, the relative risk of cancer (1.04) was not statistically significant. Therefore, based on their research, the existence of AIP was identified as a risk factor for cancer. Consistent with the results of this study, Hart et al. [24] reported that the risk of cancer before and after the diagnosis of acute pancreatitis was similar to that of the control group. In addition, Oh et al. [25] reported a case of cholangiocarcinoma, which was finally diagnosed as early cholangiocarcinoma under the background of sclerosing cholangitis and AIP. In that case, both cholangiocarcinoma and AIP were present. Although the histopathology confirmed an increase in IgG4-positive plasma cells in the bile duct, it was difficult to determine whether the cholangiocarcinoma was caused by AIP.

Some mechanisms have been proposed regarding whether the role of the IgG4 response in cholangiocarcinoma may help tumor cells evade immune surveillance. Wallance et al. [21] speculated that one possible mechanism is that malignant tumors trigger autoantigen expression, and the treatment of malignant tumors with radiation and chemotherapy leads to immune dysregulation and the development of IgG4-RD. Harada et al. [26] speculated that bile duct cancer cells may play the role of non-professional antigen presenting cells that promote regulatory T cells, which can directly or indirectly promote an IL-10-mediated IgG4 response. Other studies suggest that IgG4 may have anti-inflammatory properties, which can weaken the effective function of IgG1 using a variety of methods, allowing tumor cells to escape immune surveillance.

The present case can best be considered as a warning regarding the treatment of patients with AIP combined with common bile duct adenocarcinoma. For patients who fully meet one of the AIP diagnostic criteria, steroid therapy may be an option and patient follow-up should be strengthened. However, if the patient only partially meets these criteria or if the imaging abnormalities are not resolved quickly during treatment, malignant tumors should be suspected, and resection must be carefully considered.

Therefore, if non-specific morphological changes in the pancreas or bile ducts occur during IG4-associated AIP, aggressive pathologic examination and regular patient follow-up are required to detect a potential malignancy at an early stage.

## Data Availability

Not applicable.

## Abbreviations

AIP: Autoimmune pancreatitis
IgG4-RD: IgG4-related disease
IAC: IgG4-associated cholangitis
EUS-FNA: Endoscopic ultrasonography guided fine needle aspiration
PTCD: Percutaneous transhepatic cholangiography and drainage
CA19-9: Carbohydrate antigen 19–9
CT: Computed tomography
MR: Magnetic resonance
PET/CT: Positron emission tomography-computed tomography
HPF: High power field
